# Recent Advances in Understanding the Role of Autophagy in Paediatric Brain Tumours

**DOI:** 10.3390/diagnostics11030481

**Published:** 2021-03-09

**Authors:** Francesca Gatto, Giacomo Milletti, Andrea Carai, Angela Mastronuzzi, Francesca Nazio

**Affiliations:** 1 Department of Pediatric Hemato-Oncology and Cell and Gene Therapy, Bambino Gesù Children’s Hospital, IRCCS, 00165 Rome, Italy; francesca.gatto@opbg.net (F.G.); giacomo.milletti@opbg.net (G.M.); angela.mastronuzzi@opbg.net (A.M.); 2 Department of Biology, University of Rome ‘Tor Vergata’, 00133 Rome, Italy; 3 Department of Neurosciences, Bambino Gesù Children’s Hospital, IRCCS, 00165 Rome, Italy; andrea.carai@opbg.net

**Keywords:** autophagy, brain tumours, targeted therapy, oncology

## Abstract

Autophagy is a degradative process occurring in eukaryotic cells to maintain homeostasis and cell survival. After stressful conditions including nutrient deprivation, hypoxia or drugs administration, autophagy is induced to counteract pathways that could lead to cell death. In cancer, autophagy plays a paradoxical role, acting both as tumour suppressor—by cleaning cells from damaged organelles and inhibiting inflammation or, alternatively, by promoting genomic stability and tumour adaptive response—or as a pro-survival mechanism to protect cells from stresses such as chemotherapy. Neural-derived paediatric solid tumours represent a variety of childhood cancers with unique anatomical location, cellular origins, and clinical presentation. These tumours are a leading cause of morbidity and mortality among children and new molecular diagnostics and therapies are necessary for longer survival and reduced morbidity. Here, we review advances in our understanding of how autophagy modulation exhibits antitumor properties in experimental models of paediatric brain tumours, i.e., medulloblastoma (MB), ependymoma (EPN), paediatric low-grade and high-grade gliomas (LGGs, HGGs), atypical teratoid/rhabdoid tumours (ATRTs), and retinoblastoma (RB). We also discuss clinical perspectives to consider how targeting autophagy may be relevant in these specific paediatric tumours.

## 1. The Autophagy Process

Autophagy is an evolutionarily conserved process that mediates the degradation of cellular components; this process is driven by a set of specific genes, named Autophagy-related Genes (ATGs). Autophagy occurs in all eukaryotic cells at low basal levels to preserve cellular homeostasis through the digestion of potentially harmful intracellular elements, such as misfolded proteins, damaged or senescent organelles and aggregates [1,2]. The autophagy flux can be induced by specific changes in the intra- and extracellular microenvironment (such as lack of nutrients, hypoxia, immune signals), thus representing one of the mechanisms responsible for the cellular adaptation to stress conditions [3]. In this context, it is therefore reasonable to assume that autophagy impairment could contribute to human diseases such as neurodegenerative disorders, cancers or viral infections [4,5,6]. Although the exact mechanisms underlying the autophagy-associated diseases at the molecular level remain not fully elucidated, the recent improvements in autophagy investigation show a potential target for its manipulation in human pathologies.

In mammals, autophagy is classically divided into three main categories: macroautophagy (hereafter referred to simply as autophagy), microautophagy, and chaperone-mediated autophagy, all of which culminate in the lysosomal degradation and recycling of a particular cargo [7]. The former is the most extensively studied and will be the focus of this review, with a particular emphasis on the role it plays in paediatric brain tumours.

**Figure 1:**
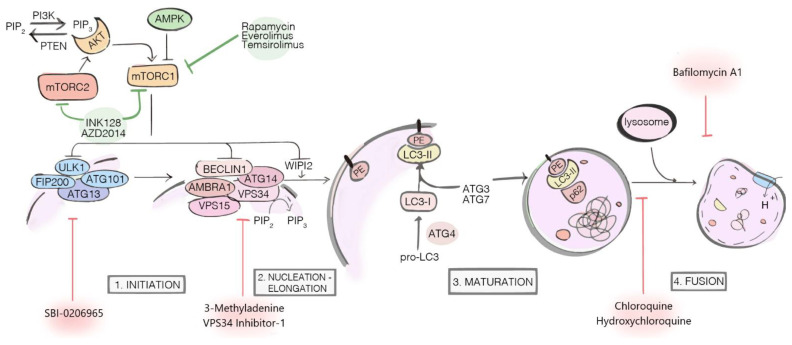
Major components and regulators of the autophagy machinery. The autophagy inhibitors (red arrows) exert their actions at different stages of the process: SBI-0206965, 3-Methyladenine and VPS34 Inhibitor-1 are early-stage inhibitors, blocking autophagy before the autophagosome nucleation; Chloroquine (CQ), Hydroxychloroquine (HCQ) and Bafilomycin A1 are late-stage inhibitors, respectively blocking the autophagosome fusion with lysosome (CQ and HCQ) and inhibiting lysosomal acidification (Bafilomycin A1). The autophagy inducers (green arrows) Rapamycin, Everolimus, Temsirolimus, INK128, and AZD2014 target and inhibit mTOR1/2 complexes, indirectly activating autophagy.

The autophagy machinery includes several protein complexes, which are responsible for the nucleation of double-membrane vesicles, called autophagosomes, encapsulating the cellular components targeted for degradation. After their formation, autophagosomes fuse with lysosomes, ultimately leading to the digestion and the subsequent recycling of their content [8]. Despite being apparently easy to sum up, autophagy is a complex mechanism, subject to a fine-tuned regulation in each step to ensure the proper functioning of the whole machinery [9,10]. Briefly, the most important protein complexes involved in the autophagosome biogenesis include:

- the Atg1/ULK complex—required in the initiation step;
- the Beclin 1 core complex—a class III PI3K complex, required for the vesicles’ nucleation phase;
- two ubiquitin-like conjugation systems (ATG12 and ATG8/LC3 conjugation complexes) and several other core proteins, including ATG9 and p62/SQSTM1, the latter functioning as an “autophagy receptor” involved in the specific targeting of cargos [8,11,12,13] (Figure 1).

Autophagy can either be a selective or a non-selective process, depending on whether or not it specifically targets a particular intracellular entity for degradation [14]. The selectivity relies on the existence of the so-called specific autophagic receptors (SARs), which are generally characterized by both ubiquitin-binding domain (UBD) and LC3-interacting region (LIR); thus, they act as adaptor proteins between the cargo and the autophagosome membrane [15]. Examples of SARs include Bcl-2 19 kDa interacting protein 3 (BNIP3), BNIP3-like (BNIP3L), neighbour of BRCA1 gene 1 (NBR1), nuclear dot protein 52 kDa (NDP52), and optineurin (OPTN) [16,17,18,19]; each of them selectively targets a given cargo, such as intracellular organelles (mitophagy, nucleophagy, pexophagy, ER-phagy), invading pathogens (xenophagy) or molecular aggregates (aggrephagy).

The best characterized upstream regulator of autophagy is the mechanistic target of rapamycin complex 1 (mTORC1), a serine/threonine kinase functioning as an autophagy inhibitor through direct targeting of several autophagy-related proteins [10]. mTORC1 is a nutrient sensor activated downstream of the PI3K/AKT pathway through metabolic inputs; deregulation of the mTORC signalling pathway, which is common in many cancers [20], renders its pharmacologic targeting of particular interest within the context of investigating the role of autophagy in tumours [21].

## 2. Role of Autophagy in Cancer: Tumour Suppression vs. Tumour Promotion

The role of autophagy in cancer has long been discussed, still being at the centre of the scientific debate [22,23]. It is now well known that autophagy can act either as a tumour-suppressor or as a tumour-promoter mechanism, depending on the specific context [24,25,26,27]. In healthy or premalignant cells, autophagy represents one of the major mechanisms contributing to the prevention of transformation by means of degrading potentially oncogenic molecules and maintaining intracellular homeostasis [28], e.g., preventing oxidative stress and DNA damage, which are known causes of cancer initiation and progression [29]. Mice models of autophagy disruption show an increase in spontaneous tumorigenesis, as observed within the first such model where there is heterozygous ablation of *Beclin 1* gene [30]. Similarly, tissue-specific knockdown of *Atg7* and mosaic deletion of *Atg5* in mice cause the development of liver adenomas [31]. Taken together, these findings show that autophagy impairment can act as a strong oncogenic driver in healthy cells and tissues. By contrast, autophagy is proved to be a survival mechanism when it comes to already established tumours [32,33], therefore representing a promising target for therapeutics. Of note, autophagy helps tumour cells maintain energetic balance and the proper redox status, thereby favouring their survival and sustaining tumour progression [26]. A huge variety of studies enlightening the tumour-promoter role of autophagy have been conducted using genetically engineered mouse models [34,35,36]; all of them shed light on the possibility to manipulate autophagy for therapeutic intervention.

Other than its controversial role in tumorigenesis, autophagy is known to be involved in chemoresistance: being a cytoprotective mechanism of adaptation to stress conditions, autophagy often mediates treatment resistance in cancer cells, thus representing an obstacle to chemotherapy [37,38,39]. The mechanisms by which this happens are certainly multifaceted and stimuli-dependent, with most of them remaining yet to be fully elucidated. Very recently, it has been proposed that exosome release within the tumour microenvironment (TME) can mediate autophagy-dependent therapy resistance [40]. Additionally, cancer stem cells (CSCs) are also known to be significantly involved in autophagy-mediated chemo- and immune-resistance, combining their already aggressive phenotype to autophagy activation, which further enhances tumour drug-resistance [41,42,43,44,45]. In this perspective, the blockade of autophagy can re-sensitize cancer cells to chemotherapeutic agents, thus enhancing the cytotoxic activity of standard therapies and powerfully overcoming chemotherapy resistance [39,46]. On the other hand, it is also known that, in certain circumstances, autophagy is able to trigger apoptosis (in the so-called “autophagy-mediated cell death”) [47,48] in the tumoral mass, thus representing a process which is worth exploiting to boost standard therapy.

Emerging studies are investigating the role of epigenetics in the modulation of autophagy in cancers. Both DNA methylation and histone acetylation/methylation are involved in the regulation of the *ATGs* expression, either favouring cancer cell aggressiveness or promoting tumour cell death [49]. Additionally, miRNAs play an important role as epigenetic regulators by means of impairing the protein levels of their target mRNAs or, more specifically, by targeting epigenetic-related enzymes in the so-called “miRNA epigenetic feedback loop” [50,51]. Among these, miR92b and miR101 negatively modulate the expression of enhancer Of Zeste 2 Polycomb Repressive Complex 2 Subunit (EZH2), a histone-lysine N-methyltransferase that can favour or inhibit autophagy depending on the tumour models; these aspects have been recently reviewed by Peixoto et collaborators [49]. 

To date, given the dual and often controversial role of autophagy depending on the tumour stage, type and microenvironment, both autophagy inhibitors and activators have been proposed as potential pharmacological strategies to treat cancer (Figure 1). Detailed descriptions about the currently available pharmacological compounds acting on autophagy—either promoters or inhibitors—together with the up-to-date clinical trials on tumours were reviewed elsewhere [52].

Here, we focus on the most recent discoveries in the context of autophagy-dependent mechanisms driving paediatric brain tumour survival and progression, together with the strategies that can be used to overcome autophagy-mediated drug resistance. This is of great importance in paediatrics because of the pronounced impact of long-term side effects occurring in children in response to therapies—i.e., neuroendocrine dysfunctions and impaired cognitive functions—compared to the adult counterpart [53,54,55]. 

## 3. Paediatric Central Nervous System Tumours

**Figure 2:**
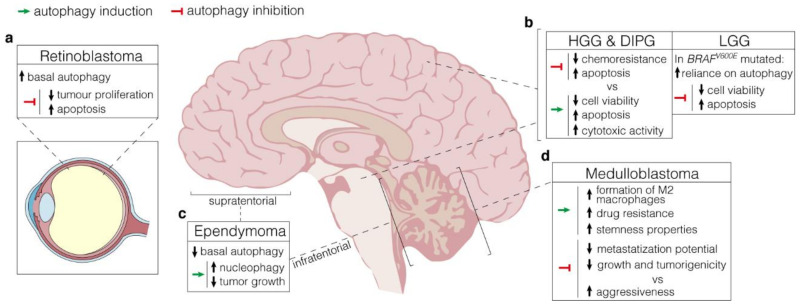
Autophagy manipulation as a therapeutical strategy. (**a**). Retinoblastoma displays high protein expression of both LC3 and p62, which supports elevated basal levels of autophagy. Autophagy inhibition by lncRNA XIST targeting negatively affects tumour proliferation while increasing apoptosis; (**b**), LEFT, In HGG and DIPG, arising in the cerebral hemispheres and the pons respectively, autophagy manipulation results in controversial outcomes. Both activation (through the administration of a range of mTOR inhibitors) and inhibition (by 3-MA) have beneficial effects on cytotoxic activity and apoptosis, with detrimental consequences on chemoresistance. RIGHT, In *BRAF^V600E^* LGG, high autophagy levels have a pro-survival role. Hence, its inhibition through either pharmacologic or genetic approaches promotes apoptosis and reduces cell viability; (**c**), Supratentorial and infratentorial ependymomas are characterized by low basal levels of autophagy. Stimulation through rapamycin promotes nucleophagy and negatively affects tumour growth; (**d**), In medulloblastoma, autophagy induction via hypoxic conditions favours drug resistance and stemness properties through the formation of M2 macrophages in TME. Depending on the context, however, autophagy inhibition produces conflicting effects, either reducing metastasization potential and tumorigenicity or promoting aggressiveness.

Tumours deriving from the central nervous system (CNS) are amongst the leading causes of cancer-related deaths in children. Surgery, aggressive radiation, and patient-stratified chemotherapy have improved the therapeutic outcomes, but too many patients still die from these diseases. Moreover, the fragility and inability of nervous tissues to efficiently regenerate, leave those who survive with devastating long-term therapy-related side effects, ranging from neuro-cognitive to motor control deficits [53,56,57]. Only recently, integrated multi-omics analyses in combination with single-cell transcriptomic clustering have provided extraordinary insights into both the inter- and the intra-tumoral heterogeneity characterizing these malignancies. By means of these approaches, it is now possible to elucidate the developmental trajectories underlying tumour onset, thereby identifying a specific sub-population of glial and neural stem cells as their cell-of-origin [58]. The most common malignant paediatric primary brain tumours are medulloblastoma, ependymoma, diffuse intrinsic pontine glioma, atypical teratoid/rhabdoid tumour, and retinoblastoma. We present the current state of the art in the molecular classification and the clinical features for most of the neural-derived paediatric tumours, and we discuss the role of autophagy-related mechanisms in each of these contexts (Figure 2).

## 4. Autophagy in Childhood Brain Tumours

### 4.1. Medulloblastoma (MB)

MB is an embryonal tumour of the cerebellum and it is considered to arise from cells in the extracerebellar lower rhombic lip or from cerebellar granule neuron progenitors (GNPs) that reside in the external granular layer (EGL) [59]. Depending on the genetic alterations, the current consensus divides MB into four main subgroups: WNT, SHH (sonic hedgehog), Group 3, and Group 4, each showing clinically and molecularly relevant peculiarities. Insights into genetic modifications in rare hereditary syndromes such as Gorlin syndrome (GS) (mutations in *PTCH1* and *SUFU*), Li–Fraumeni syndrome (LFS) (mutations in *TP53*), and familial adenomatous polyposis (FAP) syndrome (mutations in APC), provided an evident causative relationship between the mutated genes and the pathways involved in MB pathogenesis, sharpening their molecular-based classification [60]. For instance, germline mutations in *PTCH1* or *SUFU* (negative regulators of the SHH signalling pathway) are often mutated in SHH-MB. Conversely, in a retrospective genetic study, *APC* germline mutations together with somatic *CTNNB1* mutations were identified in 97% of all WNT-driven MB [61]. By contrast, Group 3 and Group 4 genetics remained partially elusive. While *MYCN* amplifications were found with comparable low frequency, *MYC* amplification was restricted to 17% of Group 3 MB patients. Similar distribution in both subgroups was detected also for structural variants leading to the aberrant and mutually exclusive activation of growth factor independent 1 family proto-oncogenes (*GFI1* or *GFI1B*) [62]. Additionally, pathways analysis of mutational landscape revealed abnormal overrepresentation of genes involved in the Notch and TGFβ signalling pathways in Group 3, and in chromatin modification in Group 4 [63]. Recently, large-scale methylation analyses have helped in profiling novel MB sub-classes of primary importance to identify innovative patient-stratified therapeutic approaches [64].

#### Relationship between Autophagy and MB Subtypes

Only recently, several studies have been emerging regarding the contribution of autophagy in MB; however, the role of autophagy in MB aggressiveness and tumorigenesis appears far from being straightforward. By means of the inducible expression of a shRNA targeting *ATG5* in MB Group 3 and SHH subgroups, Paul and colleagues found that the inhibition of autophagy plays a significant role in modulating the tumours’ invasion potential rather than its anchorage-dependent growth [65]. The MB’ capability of metastasizing turned out to be significantly reduced in both subgroups as a consequence of autophagy impairment, thus implying that autophagy is somehow connected to the degradation of essential cellular components, most probably the focal adhesion complexes, involved in the cell motility. In this perspective, inhibition of autophagy would be an important therapeutic strategy to counteract metastasization. 

Several miRNAs are known to be deregulated in MB, with some of them playing a role in autophagy modulation [50]. Thus, one of the strategies that has recently been under investigation to inhibit autophagy in MB is the restoration of specific deregulated miRNAs. In this respect, Singh et al. found that miR-30a, which is known to inhibit autophagy by downregulating *Beclin 1* and *ATG5* expression, is under expressed in all the four MB subgroups compared to healthy brain tissue [66]. Interestingly, in this study, miR-30a restoration is proved to block autophagy as well as growth and tumorigenicity of MB. Consequently, miR-30a could be considered as a treatment approach for MB via suppressing autophagy. More recently, low expression of miR-204 in MB Group 3/4 tumours identifies a highly aggressive set of tumours [67]. Other than inhibiting MB anchorage-independent growth, invasion potential and tumorigenicity, miR-204 expression also blocks autophagy and the lysosomal degradation pathway. Intriguingly, *LC3B* is a known target of miR-204 [68]. These studies suggest a crucial role for autophagy in MB progression.

On the contrary, a huge study from Čančer and collaborators reveals that mTOR activation promotes tumour malignancy and aggressiveness in different humanized stem cell models of paediatric SHH MB, either derived from iPSC-reprogrammed neural embryonic stem cells (NES) or from embryonic hindbrain neural stem cells via MYCN lentiviral overexpression [69]. In these models, mTOR activation is dependent on both *MYCN* and *POU5F1* expression levels, meaning that its inhibition is efficient in preventing tumorigenesis when the expression of these genes is upregulated or epigenetically enhanced. The mTOR inhibitor INK128 is, in fact, able to prevent the formation of spinal cord metastases and to dramatically reduce the primary tumour size. Targeting mTOR with specific inhibitors therefore results in autophagy induction, which might lay the foundation to the hypothesis of autophagy functioning as a tumour suppressor mechanism within this context. Additional investigations are then needed to enlighten the role of autophagy in SHH MB models. Similarly, Bhoopathi et al. demonstrated that the Secreted Protein Acidic and Rich in Cysteine (SPARC) overexpression in neuroectodermal tumours induces autophagy-mediated apoptosis and enhances the efficacy of radiation therapy [70,71]. However, when combined with chemotherapy instead of radiation therapy, SPARC expression induces chemoresistance; particularly, it suppresses cisplatin sensitivity in MB cells due to the increased levels of autophagy, acting as a tumour survival mechanism [72].

In brain tumours, several studies have associated hypoxia-related genes to tumour progression and chemo-/radio-resistance [73,74]. Hypoxia has been shown to induce autophagy in different cellular models, acting as a survival mechanism for hypoxic cells through recycling of cellular constituents [75,76]. Two recent studies began to correlate hypoxia and autophagy in MB. In 2016, by analysing 41 paediatric fresh-frozen MB samples, Cruzeiro and collaborators found that HIF1-α is overexpressed in MB patients when compared to the normal cerebellum [77]. When HIF1-α is downregulated in UW402 MB cell-line (SHH subgroup), a decrease in cell viability is observed together with an increase in the methylation of *ATG16L1* promoter; ATG16L1 is a core autophagy protein implicated in distinct phases of autophagosome biogenesis [78]. Although ATG16L1 activation levels were not determined in the study, there is scientific consensus about the effect of gene promoter methylation in the transcriptional silencing of genes [79]. Overall, these observations suggest a pro-survival role of autophagy within UW402 MB cells, enlightening an epigenetic link between hypoxia and autophagy genes activation. More recently, by means of a mass spectrometry-based multi-omics pilot study of cerebrospinal fluid (CSF) from recurrent MB patients, a molecular signature indicative for hypoxia has been found [80]. The authors speculate that hypoxia induces the formation of M2 macrophages via autophagy activation, stimulating a metabolic shift that supports the development of drug resistance and staminal properties of MB cells. These studies imply an involvement of hypoxia-induced autophagy in MB; however, more studies are necessary to dissect this point.

Since each potential pharmacologic target may regulate several pathways and have numerous contradictory roles within the cell, caution should be taken when choosing potential strategies for targeting autophagy.

### 4.2. Atypical Teratoid Rhabdoid Tumour (ATRT)

Atypical teratoid/rhabdoid tumours (ATRT) are rare malignant CNS embryonal tumours typically occurring in children aged 3 or younger. Despite being molecularly well-characterized, the aggressive growth and propensity for precocious metastatic spread throughout the neuroaxis confers to ATRT a dismal prognosis with an overall median survival of 17 months [81,82]. The genetic hallmark of ATRT is the biallelic loss-of-function mutation in SWI/SNF-related, matrix-associated, actin-dependent regulator of chromatin subfamily B member 1 (*SMARCB1*), a constitutive component of the SWI/SNF chromatin-remodelling complex. In very few cases, *SMARCB1* alterations are replaced by other SWI/SNF complex members such as *SMARCA4* [83]. Recently, two independent publications have classified ATRTs into three epigenetic subtypes in which specific lineage-enriched methylation and transcriptional signatures correspond to distinct clinical features [84,85]. Analysis of the upregulated pathways have provided a consensus nomenclature for each of them: ATRT-TYR, ATRT-SHH, and ATRT-MYC. Of note, each group possesses a preferential onset location, possibly implying that they may originate from different precursor cells. Respectively, ATRT-TYR tumours are defined by overexpression of several melanosomal markers, such as *MITF*, *TYR* or *DCT*; the SHH subgroup is characterized by the overexpression of *MYCN*, *GLI2* (SHH signalling targets) and by NOTCH signalling upregulation; ATRT-MYC is named after the marked increase in MYC expression [86]. Despite the significant prognostic value of this subgrouping, major efforts are still ongoing to identify effective targeted therapies.

#### Role of Autophagy in ATRT

A combinatorial strategy of autophagy modulation using rapamycin or chloroquine (CQ) was investigated by Levy and Thorburn in BT-16+ BT-12 CNS atypical teratoid/rhabdoid tumour cells in combination with chemotherapy drugs 1-(2-chloroethyl)-3-cyclohexyl-1-nitrosourea (CCNU) and cisplatin [87]. As in many other tumours, it was observed that CCNU and cisplatin cause the induction of autophagy in these cells; however, combinations do not significantly increase the chemotherapeutics efficacy. Previously, Watanabe and colleagues found that the histone deacetylase (HDAC) inhibitor FK228 induces autophagy in malignant ATRT cells by favouring apoptosis inducing factor (AIF) translocation to the nucleus [88]. HDAC inhibitors are, indeed, known to potentiate the effect of ionizing radiation on ATRT cells [89] and to promote autophagy cell death [49], strengthening the role of epigenetics on autophagy modulation. These results indicate that autophagy inhibition is not as straight-forward as initially wished and cell response to modulation is probably to be considered context-dependent. Moreover, these findings lead the way to the use of epigenetic drugs (epidrugs) as a co-treatment in cancer therapies. 

### 4.3. Low-Grade Glioma (LGG)

In paediatric age, the vast majority of intra-axial brain tumours arising from the glial lineage are low-grade gliomas (LGGs) [90] with simple molecular alteration [91] and low rate of malignant transformation (around 7%) [92]. These tumours are essentially driven by a single genetic aberration, typically affecting the MAPK pathway [93]. The most common mutation observed in LGG is the well-characterized BRAF V600E alteration [94]. BRAF is a serine-threonine MAPKKK involved in the RAS/RAF/MEK/ERK signalling pathway [95], which is implicated in a wide range of cellular functions including proliferation, differentiation, and apoptosis; mutations of the BRAF gene are very common in several types of cancer [96]. Particularly, the BRAF V600E mutation causes the constitutive activation of the MAPK pathway, leading to carcinogenesis. The second most common point mutation occurs in the kinase domain of FGFR1 [97].

#### Autophagy Dependence for Therapy Resistance in LGG

As reported above, BRAF mutations are common in gangliogliomas, pleomorphic xanthoastrocytoma, and cerebral pilocytic astrocytomas [98]. BRAFV600E mutation has been found to increase the tumours’ reliance on autophagy, thereby increasing their susceptibility to autophagy inhibitors compared to their wild-type counterparts [99,100]. Specifically, MAF794 cell line, derived from a paediatric ganglioglioma carrying BRAF V600E mutation, shows higher autophagy levels when compared to BT16 BRAF wild-type cells; moreover, autophagy inhibition—either pharmacologic or genetic—leads to cell viability reduction. Combination therapy of chloroquine—a late-stage autophagy inhibitor—with cisplatin and vinblastine is even more efficient in tumour killing compared to CQ alone; by contrast, CQ does not increase the tumour cell death in BT16 cells [100]. Additionally, using both pharmacologic (ULK1 inhibitor SBI-0206965 and VPS34 inhibitor-1) and genetic inhibition of early-stage autophagy regulators, together with the standard BRAF-inhibitors (Vemurafenib), Zahedi et al. demonstrated an ameliorated response compared to the administration of each treatment alone, resulting in the activation of an apoptotic cell death [99]. Because BRAF mutation is generally more common in paediatric CNS tumours than in adult cases, the strategy of targeting autophagy holds promises for this group of tumours.

### 4.4. High-Grade Glioma (HGG) and Diffuse Intrinsic Pontine Glioma (DIPG)

Although rare, paediatric high-grade gliomas (pHGG) and Diffuse Midline Gliomas (DMG, including diffuse intrinsic pontine glioma—DIPG) retain both the dramatic prognosis and the canonical deregulated tumour pathways [(RTK)– RAS–PI3K, p53 and RB)] that arise in adults [101]. However, when compared with their adult counterparts, pHGGs emerge in different locations and show distinctive mutated effectors with the platelet-derived growth factor receptor-α (PDGFRA) being the most commonly altered RTK instead of the epidermal growth factor receptor (EGFR) [102]. Consistently, while isocitrate dehydrogenase 1 (IDH1) or IDH2 are highly mutated in adult HGG [103], these enzymes are frequently unaffected in pHGG. Mutations of *TP53* are found in approximately 55% of pHGGs [104,105], with specific mutually exclusive mutations in 9–23% of DIPGs and midline HGGs in the *TP53* target gene *PPM1D*. Focal amplification of cyclin-dependent kinase 4 (CDK4), CDK6 or CYCLIN D1 (CCND1), CCND2, or CCND3 respectively, that impact Rb phosphorylation status, are present in pHGGs from all brain regions [104]. Accounting for almost one-half of all pHGG, DIPG is a brainstem-located tumour that occurs almost exclusively in children. High-throughput genome-wide analysis in multiple cohorts of pHGG and DIPG identify unique recurrent mutations in histones H3.3 and H3.1 [106,107], respectively encoded by (*H3F3A*) and histone cluster 1, H3b (*HIST1H3B*). As a confirmation of the distinctiveness underlying the paediatric disease, these mutations are not found in the adult HGG. Histone H3 mutations occur at two residues, the lysine at position 27 [(lysine to methionine (K27M)] and the glycine at position 34 [glycine to arginine (G34R) or glycine to valine (G34V)], with the former accounting for almost 80% of DIPG cases and the latter predominantly occurring in tumours of the cerebral hemispheres. After H3F3A and TP53, the next most frequently mutated gene in DIPG is activin receptor type 1 (*ACVR1*) [104,108,109], which encodes for a bone morphogenetic protein (BMP) receptor that activates SMAD signalling cascade. Despite the detailed molecular insights obtained through integrated genomic analyses, no targeted therapy or chemotherapy has provided a survival benefit for pHGG patients when administered alone or in combination with other drugs; hence, the urgency of novel therapeutic approaches.

#### The Contradictory Roles of Autophagy in HGG and DIPG

In recent years, it has become increasingly clear that HGGs and DIPGs in children differ on a molecular basis from HGGs in adults, and many of the molecularly targeted strategies that have been employed based on adult data have little applicability in the paediatric context. The role of autophagy in adults, especially in glioblastoma, has been extensively studied and recently reviewed by Escamilla-Ramírez et al. [110]. Particularly, its role has mainly been investigated in regard to its contribution to the induction of chemoresistance. In this respect, a study showed that the cytotoxic action of temozolomide (TMZ), an alkylating agent used as a first line chemotherapeutic for gliomas, is at least in part related to an autophagy response in adult glioblastoma, followed by apoptosis and senescence [111]. All these effects are prevented by the expression of O^6^-methylguanine-DNA methyltransferase (MGMT) by tumour cells: indeed, the trigger for both the autophagic and the apoptotic responses via ataxia-telangiectasia mutated protein kinase (ATM) activation is represented by the O^6^MeG TMZ-induced DNA lesion. Similarly, the MGMT promoter methylation, which is responsible for the gene silencing, in paediatric glioblastoma is correlated with a longer survival in TMZ-treated patients [112]. Within this context, the MGMT promoter methylation might act as an epigenetic mechanism that indirectly regulates autophagy. Moreover, autophagy was proven to be a pro-survival mechanism for the tumour, mediating chemoresistance and preventing apoptosis: the administration of 3-methyladenine (3-MA), an autophagy inhibitor, increases the levels of apoptosis and tumour cell death [111]. In light of this, autophagy inhibition represents a strategy which, in combination with TMZ, might ameliorate the killing effect of chemotherapy.

Several miRNAs have been shown to exert an epigenetic control of autophagy in both adult and paediatric HGGs [50,113]. Comincini and collaborators first showed that miR-17 has the ability to inhibit autophagy in adult glioblastoma cells via negatively regulating *ATG7* expression [114]. This data is consistent with the later findings of Miele and collaborators showing an overexpression of miR-17-92 cluster in pHGG, which is believed to exert a quenching function on its target gene *PTEN* [115], thereby indirectly influencing autophagy in a negative fashion. 

Silva et al. found that the combination of euphol, a tetracyclic triterpene alcohol with TMZ, has the highest cytotoxic effect in paediatric glioma with respect to adult cells. This combination inhibits cell proliferation and migration by inducing autophagy-mediated cell death. However, Euphol cytotoxicity is enhanced when it is administered together with the autophagy inhibitor Bafilomycin A1, through a mechanism which has not yet been studied [116].

Howarth et al. performed for the first time an analysis on the expression levels of autophagy-related proteins in glioma patients, prior and after specific pharmacological treatments. Interestingly, it was observed that both Beclin 1 and LC3 A/B protein levels are significantly higher after the administration of an aggressive chemotherapeutic regimen of combined drugs (vincristine, platinum compounds namely carboplatin and cisplatin, methotrexate and cyclophosphamide) [117]. This observation provides us with the evidence that autophagy functions as an important mechanism of chemoresistance in pHGG: its targeting would therefore represent a strategy for attenuating drug resistance and enhancing tumour killing, especially in relapsed diseases, which are the most difficult to eradicate. Furthermore, the authors speculate that targeting autophagy may be beneficial for the entire pHGG cohort, without subgroups distinctions, because autophagy is a shared pathway in all the pHGG subgroups. Moreover, targeting autophagy may function even better if combined with subgroup-specific therapy, hence representing a promising approach to overcome drug resistance in pHGG. The exact role autophagy plays in pHGG continues to be widely debated. Information about the role of autophagy in DIPG is currently lacking; nevertheless, several studies have been conducted on DIPG after mTOR inhibition, which still leaves the door open to speculations about potential autophagy roles within this context. Tsoli et al. have observed a significant increase in cytotoxic activity, mitochondrial dysfunction, and apoptotic cell death on patient-derived DIPG cells following treatment with dual pharmacological inhibition of adenine-nucleotide translocase (ANT) mitochondrial protein (using PENAO) and mTOR (using temsirolimus) [118]. Similarly, Asby et al. demonstrated an anti-proliferative effect and a decrease in cell-viability after administration of temsirolimus, both alone and together with a CDK 4/6 inhibitor [119]. Another study, conducted by Flannery et al., evaluated the inhibition of mTORC1, both alone (everolimus) and together with mTORC2 inhibition (AZD2014) on DIPG cell lines, observing that the inhibition of both complexes via AZD2014 works better than everolimus in the elicited antitumour activity [120]. Altogether, these findings show that mTOR signalling inhibition brings along antitumor effects. With mTOR being a negative regulator of autophagy, we might hypothesize a pro-death role of autophagy in DIPG. It is worth investigating this aspect given the great potential of combined therapies for DIPG: hypothetically, an autophagy activator in association with conventional therapy could be a feasible alternative strategy to ameliorate patients’ prognosis.

### 4.5. Ependymoma (EPN)

Ependymoma (EPN) is a neuroepithelial malignancy that can arise all along the neuroaxis, including the supratentorial region (ST), the posterior fossa (PF), and the spinal cord [121]. In paediatric age, 90% of ependymomas occur intracranially, with two-thirds located in the PF and the remaining one-third within the supratentorial compartment [122]. EPNs possess a highly variable clinical behaviour that renders the identification of effective treatment extremely challenging. Consistently, 10-year overall survival (OS) is about 64% in paediatric patients [101,123,124].

By exploiting DNA methylation profiling, EPNs have been classified in nine subgroups, three per anatomical compartment of the CNS where they occur. Examination of clinical and demographic data showed that the vast majority of high-risk patients were restricted to just two of the nine molecular subgroups identified, particularly the ST-EPN-RELA that harbours *RELA* prototypic fusion, and PF-EPN-A, which instead possesses a predominantly stable genome [125]. This corroborates the clinical relevance of a stringent molecular classification.

#### Early Insights into the Role of Autophagy in EPN

The role of autophagy in ependymoma has not been specifically investigated so far. However, a study concerning the role of nucleoporin TPR upregulation within this tumour attracted the interest of researchers in regard to its connection with autophagy, given the existing correlation between TPR depletion and autophagy induction in HeLa cells [126]. By analysing mRNA expression levels of autophagy-related proteins, Dewi’s research team found that *ATG3*, *ATG5*, *ATG12,* and *Beclin 1* expression is significantly downregulated in ependymoma patients [127]. These data are confirmed by the analysis of both LC3 and p62 at the protein level, supporting an inhibition of autophagy process. Intriguingly, TPR knockdown is able to restore the levels of these genes and, broadly, is proved to induce autophagy. Particularly, TPR silencing induces nuclear membrane blebbing, hypothesizing that high levels of TPR could prevent the so-called nucleophagy—i.e., selective autophagy degradation of nuclear components [128]—favouring tumorigenesis in ependymoma. Similarly, in a xenograft mouse model, the downregulation of TPR prevents tumour growth [127]. Together with the observation that mTOR inhibitors are able to restore nucleophagy and to reduce tumour growth in vivo, these findings suggest that nucleophagy needs to be upregulated in order to lower the tumorigenic potential of ependymoma. In this perspective, autophagy induction might be a potential therapeutic approach for ependymoma patients.

### 4.6. Retinoblastoma (RB)

Retinoblastoma is the most diffused eye cancer in paediatric patients. It predominantly arises from biallelic mutation of the retinoblastoma (*RB1*) gene in a susceptible developing retinal cell. Approximately 8,000 new cases are diagnosed every year; when early detected, the tumour is commonly visible as a white mass through the pupil [129]. Prompt treatment directly correlates with a favourable prognosis, but when it is diagnosed at a later stage, incurable invasion of the optic nerve and brain may occur, making essential an extensive and invasive intervention. *RB1* complete inactivation is necessary but not sufficient to induce malignant transformation [130]. Induction of histone H3 Lys4 (H3K4) trimethylation and H3K9 and H3K14 acetylation, copy-number alterations of the mitotic kinesin family member 14 (*KIF14*) and the p53 regulator *MDM4*, expression of the spleen tyrosine kinase (*SYK*) oncogene, and loss-of function mutations in the transcriptional co-repressor BCL-6 (*BCOR*) have all been described as putative malignant effectors in RB [131,132]. Although multiple genetic and epigenetic alterations have been identified in retinoblastoma, we are still far from a comprehensive molecular classification.

#### Non-Coding RNAs-Dependent Autophagy Modulation in RB

The role of autophagy in RB tumorigenesis is not clear. Immunohistochemical analyses of RB tissues shows elevated expression levels of two autophagy-related proteins, LC3B and p62, in more than a half of the analysed samples, with their expression being significantly associated with late TNM stage and optic nerve invasion, two clinicopathological parameters of malignancy in RB [133]. Additionally, the cytoplasmic levels of p53, which is known to inhibit autophagy and induce apoptosis [134], inversely correlate with LC3B and p62 levels [133], suggesting that high levels of autophagy in RB play an important role in tumour progression.

Several miRNAs and long non-coding RNAs (lncRNAs) are deregulated in RB and most of them serve as epigenetic regulators in RB onset and development. Some of the highly expressed non-coding RNAs (ncRNAs) have been implied in RB tumorigenesis via positive autophagy modulation. Among these, MALAT1—an oncogenic lncRNA—promotes RB cell autophagy by inhibiting miR-124 expression, which, in turn, causes syntaxin 17(STX17)—an essential autophagosomal SNARE protein—overexpression [135]. Under normal conditions, miR-124 directly binds to the 3′UTR of STX17, consequently reducing its levels [135] and impairing the fusion between the autophagosome and the lysosome. Furthermore, miR-320 was demonstrated to be highly expressed in RB [136] where it favours autophagy activation via HIF1-α targeting. Particularly, under hypoxic conditions, miR-320 positively regulates both the mRNA and the protein levels of HIF1-α, which, in turn, activates autophagy [137]. Similarly, lncRNA XIST promotes autophagy in RB tumour cells. XIST silencing suppresses autophagy, tumour proliferation, and enhances apoptosis, additionally sensitizing RB cells to vincristine [138], the efficacy of which is often limited because of chemoresistance. In this respect, it has recently been discovered that CD24, a plasma membrane GPI-anchored protein being overexpressed in RB and representing a poor prognostic factor in several tumours including RB [139], impairs RB sensitivity to vincristine by means of promoting autophagy activation [140]. Particularly, CD24 is responsible for the recruitment of phosphatase and tensin homolog (PTEN) to lipid rafts, which is then able to convert phosphatidylinositol 3,4,5-trisphosphate (PIP3) to PI(4,5)P2, thus inhibiting the activation of PI3K/AKT/mTOR pathway and promoting autophagy. CD24 knockdown in RB decreases autophagy levels and restores RB sensitivity to vincristine in xenografted mouse models; similarly, treatment with chloroquine restored CD4-induced lowered response to vincristine, both in vitro and in vivo [140].

## 5. Perspectives in Clinical Research

While the combination of surgery, radiotherapy, and chemotherapy has improved survival for several types of brain tumours (e.g., medulloblastoma), other types of brain cancers (e.g., HGG and diffuse midline glioma) still retain a poor prognosis [141]. The manipulation of autophagy as a death mechanism in tumour cells has led to the use of autophagy inhibitors and inducers to counteract cancer progression [142]. Evidence of autophagy modulation in paediatric brain tumours is limited and, as in adults, its effects appear to be tumour-specific; however, its manipulation remains an exciting candidate strategy for treatment. Before such new therapies could be ultimately used in the clinical practice, their effects must be evaluated in clinical trials to support their real efficiency. The current ongoing clinical trials studying the role of autophagy inhibition in paediatric patients are still limited in number (Table 1). A phase I/II trial is investigating the side effects, best dose and efficacy of hydroxychloroquine (HCQ)—a less toxic derivative of chloroquine—in combination with dabrafenib and/or trametinib. Eligibility criteria for the study include children with LGG and HGG with a BRAF aberration, which were previously treated with similar drugs but did not respond completely or underwent recurrence (NCT04201457). Evidence of a patient being successfully treated with CQ after failure of vemurafenib suggests that autophagy inhibition might represent a powerful strategy for treating BRAFV600E-resistant brain tumours [143]. As a matter of fact, treatment with chloroquine or hydroxychloroquine is already well established in malarial infections, systemic lupus erythematosus, and rheumatoid arthritis, where these drugs are generally well tolerated and rarely cause severe side effects [144]; however, detailed information about best dosage in children is lacking and long-term administration in children is not recommended, even though a study using long-term HCQ treatment has been reported [144]. Additional trials are inevitably required to assess the efficacy and the tolerability of these drugs in paediatric patients, especially in tumoral contexts.

As mentioned above, in some tumoral paediatric contexts, autophagy induction is also being investigated as a therapeutic strategy (Table 1). The use of temsirolimus, a lipid-soluble rapamycin analogue inhibiting mTORC1 and inducing autophagy [145], in combination with valproic acid has been investigated in a recent phase 1 clinical trial (NCT01204450) in highly pre-treated paediatric patients with refractory solid tumours (central nervous system tumours, neuroblastoma, sarcoma). Moreover, some studies are testing the use of everolimus alone (NCT00187174) or in combination with lenvatinib (NCT03245151) in recurrent and refractory paediatric solid tumours, including CNS tumours. Everolimus, a rapamycin derivative, is an orally administered mTOR inhibitor with activity against various tumour types, including glioblastoma [146].

Since it is not obvious in distinguishing to what extent autophagy results in a positive or negative equilibrium for cell survival, it is highly important to act on this process in a specific manner. It is therefore crucial to consider the molecular, cellular, and epigenetic context as well as the heterogeneity that characterize these tumours. The choice between inhibiting or favouring autophagy as a co-treatment should then be based on the specific drug, which is being used for therapy: care must be taken when evaluating the therapeutic effects of a drug, considering whether determined, drug dependent, autophagy activation or abrogation might be selectively regulated by existing anti-autophagic compounds in order to define more effective tailored therapies.

## Figures and Tables

**Table 1:** Clinical trials using autophagy inhibitors/activators in paediatric brain tumours.

Clinicaltrials.Gov ID	Study Phase	Treatment	Autophagy Modulation	Pathologic Condition(s)			
NCT04201457	Phase I/II	Hydroxychloroquine + Dabrafenib	Inhibition	paediatric Low-Grade Glioma (pLGG) with BRAF Aberration			
		and/or Trametinib		paediatric High-Grade Glioma (pHGG) with BRAF Aberration			
				pLGG of Brain with Neurofibromatosis Type 1			
NCT01204450	Phase I	Temsirolimus + Valproic Acid	Induction	Brain and Central Nervous System Tumors			
				Neuroblastoma			
NCT00187174	Phase I	Everolimus	Induction	Brain Tumors			
NCT03245151	Phase I/II	Everolimus + Lenvatinib	Induction	CNS Tumors			
